# Secondary mitral regurgitation: reducing the leak, expanding the science

**DOI:** 10.1002/ehf2.13051

**Published:** 2020-10-05

**Authors:** Sam Straw, Dominik Schlosshan, Klaus K. Witte

**Affiliations:** ^1^ Leeds Institute of Cardiovascular and Metabolic Medicine University of Leeds Leeds UK; ^2^ Leeds Valve Service, Department of Cardiology Leeds Teaching Hospitals NHS Trust Leeds UK

## Introduction

Patients with heart failure with reduced ejection fraction (HFrEF) and any degree of secondary mitral regurgitation (MR) (SMR) have greater symptoms, higher hospitalization rates, and shorter longevity than those without.^1^, ^2^ However, as a group, they often have more impaired left ventricular (LV) function, are older, and have more co‐morbidities.^3^ Whilst medical and device therapies targeting the underlying pathophysiology of HFrEF reduce SMR and improve prognosis,^4^ whether treatments targeting SMR alter the course of the underlying disease is unclear and has been discussed for several decades.^5^ Furthermore, despite significant progress including one positive randomized controlled trial of edge‐to‐edge repair,^4^ there are still challenges ahead that require the science to progress before the case is closed.

## Are we accurately assessing secondary mitral regurgitation?

In this issue of the *Journal*, Ströbe and colleagues contribute important data describing the acute impact of the Carillon device, a fully transvenous annular approach, adding to the observations of longer‐term reductions in SMR and LV remodelling seen in the randomized, sham‐controlled Carillon Mitral Contour System for Reducing Functional Mitral Regurgitation (REDUCE‐FMR) trial.^6^ Secondly, and perhaps more importantly, they elegantly outline a strategy for the reproducible echocardiographic assessment of SMR using morphological and quantitative variables in the setting of HFrEF.^7^

The accurate assessment of the degree of MR seems a basic concept; however, accurate quantification, particularly in the setting of HFrEF, remains one of the greatest challenges in echocardiography. An integrated approach consisting of semi‐quantitative and quantitative parameters is recommended^8^, ^9^, ^10^ but has limited reproducibility owing to regional wall motion abnormalities, dyssynchrony, and LV remodelling, which introduce the potential for greater errors.^11^ The preferred quantitative measurement for both clinical and research applications uses the proximal isovelocity surface area (PISA). This has significant limitations including the fact that the effective regurgitant orifice area, assumed to be hemispheric, is actually elliptical, the measurement of the PISA radius is difficult, and any errors are squared in the calculation. PISA can therefore lead to implausible results^11^ and unreliable differentiation between even severe and non‐severe SMR.^12^

## How can these challenges be overcome?

Improvements in imaging techniques incorporating three‐dimensional imaging with less reliance on geometric assumptions have paralleled the advent of percutaneous intervention for MR.^13^ There has also been an increasing appreciation that accurate volumetric imaging, specifically that of the LV, could describe MR in terms of regurgitant volume (RVol) and regurgitant fraction (RF) as a product of the systolic volume of the LV (SV_LV_) and the forward stroke volume (SV forward) across the aortic valve:

$${\mathrm{SV}}_{\mathrm{LV}}=\left(\mathrm{end}-\text{diastolic}\;\mathrm{LV}\;\text{volume}\right)-\left(\mathrm{end}-\text{systolic}\;\mathrm{LV}\;\text{volume}\right)$$

$$\text{RVol}={\mathrm{SV}}_{\mathrm{LV}}–{\mathrm{SV}}_{\text{forward}}$$

$$\mathrm{RF}=\text{Rvol}/{\mathrm{SV}}_{\mathrm{LV}}$$

Quantitative volumetric methods are seldom used in clinical practice and are not actively encouraged by guidelines^10^; however, Stöbe and colleagues have demonstrated that they can be applied. More routine use of three‐dimensional echocardiography would allow more widespread implementation of this technique, which could be further complemented by multi‐modality assessment.

## Does more accurate assessment of secondary mitral regurgitation matter?

Whether one chooses to adopt such a comprehensive or a more pragmatic approach in routine clinical practice depends somewhat on the proposed treatment, its mode of action, and safety profile. For example, in contrast to the Transcatheter Mitral‐Valve Repair in Patients with Heart Failure (COAPT) trial,^4^ local valve‐teams decided upon eligibility for the annular treatment in REDUCE‐FMR.^6^ The inclusion of patients with less severe SMR as assessed by subsequent core‐laboratory analyses reduced the mean improvement in SMR achieved (improving mild SMR is difficult). However, this approach had the effect of delivering information on a wider range of patients including a group in whom, based upon the observation that any degree of SMR is associated with a worse outcome, one could envisage a future strategy of low‐risk prevention. Secondly, REDUCE‐FMR also provides reassurance to future payers, clinicians, and patients of potential outcomes in patients recruited in real‐world situations outside of core‐laboratory control. Given the adverse impact of *any* SMR on outcomes, it is plausible that a safe, simple, and preferably inexpensive procedure might be applied at a much earlier stage obviating the need for accurate assessment. But we are not at that stage yet.

## Proportionate and disproportionate secondary mitral regurgitation

Although to many, the case for mitral valve‐targeted treatments in HFrEF is closed, this strategy is based on a single randomized trial of a single technique in a highly controlled setting.^4^ The divergent results of the Percutaneous Repair of Medical Treatment for Secondary Mitral Regurgitation (MITRA‐FR) study^14^ has led to a plausible concept of proportionate SMR (appropriate for the degree of LV remodelling) and disproportionate SMR (greater than would be expected for the degree of LV remodelling),^15^ where those with disproportionate SMR might benefit more from valve‐targeted intervention. However, recent data from the COAPT study do not support a heterogeneity of effects on clinical outcomes in patients by baseline LV volume.^16^ Moreover, one cannot assume that any such concept holds for newer or less studied devices with different modes of action. For example, in COAPT, although those allocated to intervention had lesser progression of remodelling, neither group experienced reverse remodelling,^16^ a consistent observation in patients treated with the Carillon device.^17^ Finally, whether this model reflects differing pathophysiology or merely the duration of the HFrEF syndrome is unknown and will require longitudinal follow‐up of those with untreated disproportionate SMR to determine if in time they become patients with proportionate SMR. Hence, caution should be exercised when (de)selecting patients who have severe proportionate SMR despite optimal medical therapy until we have more information from studies that support this paradigm. Both groups, including those with disproportionate SMR and HFrEF, should continue to receive optimal medical therapy targeting the renin–angiotensin–aldosterone system and beta‐adrenoceptor antagonists in addition to being considered for device therapy.^13^

## Pathophysiological mechanisms and co‐morbidities guiding therapeutic approach in secondary mitral regurgitation

Although much attention is given to the assessment of the degree of SMR, the growing number of transcatheter devices offers the opportunity but also the challenge of selecting the most appropriate device, or combination of devices to treat patients with SMR based upon the pathophysiology. The mechanisms underlying SMR are heterogeneous with LV remodelling, regional wall motion abnormalities due to ischaemia, infarction, and electromechanical dyssynchrony being accepted as underlying contributors, whilst annular deformation, annular dilatation, mitral valve leaflet area, degree of leaflet tethering, and left atrial function differ significantly depending on underlying aetiology.

The status of the patient should also contribute to the choice of device through a coordinated multi‐disciplinary team approach. For example, a fully transvenous annular treatment might be more effective in patients with more adverse remodelling and could be considered in patients with co‐morbidities or with milder SMR in whom a shorter, low‐risk procedure might be preferable. On the other hand, a combined approach, although providing the greatest reduction in SMR, needs to balance risk, cost, and clinical outcomes.

Atrial functional MR (AFMR) might be an additional ideal target for the Carillon device. The prevalence of AFMR is reported to be 3–15% of patients with atrial fibrillation^18^ and 15.9% in patients hospitalized with heart failure who have preserved ejection fraction.^19^ In contrast to SMR caused by LV dysfunction and dilatation, in AFMR, LV dimensions and systolic function are normal, leaving mitral annulus dilatation as the key driver of mitral leaflet malcoaptation.

## Measuring ‘response’ to treatments for secondary mitral regurgitation

Measuring the benefit of therapies in HFrEF is difficult owing to the variable symptom burden and inexorable deterioration expected even in patients receiving optimal therapies. Because of this, patient‐orientated outcomes such as exercise capacity, symptoms, and survival cannot be reliably assessed from longitudinal data.^20^ For individual patients, staying the same and deteriorating more slowly are meaningful outcomes that are only possible to determine by comparisons with the unimplanted. The lessons learned from cardiac resynchronization therapy (CRT) are relevant.^21^ Observational studies could have the unintentional consequence of (de)selection of patients in clinical practice who have little chance of ‘response’ thereby risking undertreating a population, within which the oldest, the frailest, and those with co‐morbidities potentially have the greatest proportional gain from intervention. Moreover, the relationship between the severity of SMR and outcomes is not linear; and as demonstrated by the conflicting results of COAPT, MITRA‐FR, and surgical data, reductions in SMR do not automatically improve outcomes^4^, ^14^, ^22^ and cannot be assumed (yet) to be a reliable surrogate of patient‐orientated outcomes.

## Future outcome trials

Device therapies for SMR will only be widely adopted and incorporated into guidelines if supported by randomized controlled trials, preferably with blinding where possible to avoid differential follow‐up and nocebo effects. These trials need to be powered to avoid the pitfalls of strict entry criteria that limit generalizability and subsequent subgroup analyses. If longitudinal data must be used whilst we wait for the outcomes of further trials, the Packer clinical composite score is an adequate compromise that includes failure to deteriorate as a positive outcome, which in CRT is associated with a similar benefit on long‐term mortality as ‘response’.^23^

## Conclusions

We have made great progress, and new classifications of SMR and its pathophysiology have moved the field forward greatly, but we must be willing to ‘validate, refute or modify’ our strategy^15^ for the benefit of current and future patients.

## Conflict of interest

No conflicts of interest regarding the present paper for any of the authors.
